# First Retrospective Analysis of Robot-Assisted Radical Prostatectomy Results in Uruguay

**DOI:** 10.7759/cureus.101069

**Published:** 2026-01-08

**Authors:** Samer Jaber Di Tommaso, Raul Riveros, Roberto Puente, Martin Bertacchi, Fernando Abarzua

**Affiliations:** 1 Urology, Hospital Britanico, Montevideo, URY

**Keywords:** continence, positive margins, potency, prostate cancer, radical prostatectomy, robotic surgery

## Abstract

Background

In Uruguay, prostate cancer is currently the most frequently diagnosed cancer in males and the second leading cause of cancer death. However, the mortality rate has been decreasing due to early diagnosis and treatment. Robotic surgery has played a very important role in the surgical treatment of prostate cancer, becoming the gold standard approach in different countries of the world. Only one robotic platform has been available in Uruguay since 2011. This study aimed to evaluate the perioperative, oncologic, and functional outcomes of the initial experience with robot-assisted radical prostatectomy in Uruguay, and to contextualize these results within internationally reported outcomes.

Methodology

This retrospective study analyzed outcomes of 70 patients who underwent radical robotic prostatectomy performed by a single surgeon during the initial phase of his robotic learning curve, defined as the first 70 consecutive cases performed under direct supervision by a certified proctor between March 2015 and December 2022.

Results

We report perioperative data, pathological findings, functional and oncological outcomes, and complications. The pathology report showed a 22.85% positive margin rate (11.11% of pT2 and 44% of pT3). The mean follow-up was 38 months. Complete continence was achieved in 93.54% of cases and potency in 66.12%. The conversion rate to open surgery was 1.42%, and Clavien grade III complications occurred in 4.2% of cases. Biochemical recurrence was observed in 19.35% of patients. Patients with positive margins and pT3 disease had significantly higher biochemical recurrence rates, and Kaplan-Meier analysis demonstrated a restricted mean biochemical recurrence-free survival of 50.7 months (95% confidence interval = 45.9-55.5).

Conclusions

Our functional and oncological outcomes and complication rates appear to be within the range reported in the international literature.

## Introduction

Radical prostatectomy remains the gold standard surgical treatment for localized prostate cancer [1], and can be performed using different approaches, including open surgery, laparoscopic, and robotic surgery [2]. Over the past two decades, the robotic approach has demonstrated undisputable advantages, including ergonomics, vision (three-dimensional), mobility (articulated arms), and precision (tremor filtering), which has translated to an improvement in the learning curve of the procedure [3]. With a better understanding of the anatomical features of the radical prostatectomy surgery, and the continuous development of the minimally invasive surgery, robotic surgery has come to stay, given all the undisputable advantages it provides to the surgeon and subsequently to the patient [4]. It has also demonstrated advantages in functional results against laparoscopic surgery, such as early continence [5].

In Uruguay, prostate cancer is currently the most frequently diagnosed cancer in males, with an incidence rate of 51.2 per 100,000 men registered in 2015, and the second leading cause of cancer death [6]. Before the introduction of robotic surgery, radical prostatectomy in Uruguay was mainly performed through open and laparoscopic approaches, with limited published national experience available.

Despite all the advantages mentioned before, in Uruguay, there is only one robotic platform, and the difficulty in disseminating the technique includes accessibility and the lack of coverage for most of the population by the healthcare system. This situation leads to a lower volume of surgeries and more challenges during the training of urologists in robotic surgery. To our knowledge, this is the first historical publication of radical robotic prostatectomy (RRP) outcomes in Uruguay, including perioperative and follow-up results.

## Materials and methods

We retrospectively analyzed the collected data of 70 cases of prostate cancer treated with RRP at Hospital Británico in Montevideo, Uruguay, between March 2015 and December 2022. The platform utilized was the Da Vinci Surgical System SI. All procedures were performed by a single surgeon during the initial phase of his robotic learning curve, defined as the first 70 consecutive cases performed under direct supervision by a certified proctor. The surgeon was experienced in both open and laparoscopic surgery and holds certification in robotic surgery from Intuitive, obtained in Houston, Texas (USA).

We analyzed positive surgical margin rates and their association with pathological staging. Biochemical recurrence was defined as two consecutive prostate-specific antigen (PSA) levels ≥0.2 ng/mL. Continence and potency were assessed using patient self-reports; however, future studies should incorporate standardized questionnaires, such as the International Index of Erectile Function-5 and the Expanded Prostate Cancer Index Composite-26 urinary domain, to allow for more objective and comparable assessments. Complete continence was defined as the complete absence of urinary leakage regardless of the use of protective pads, while potency was defined as the ability to achieve and maintain an erection sufficiently rigid for penetration during sexual intercourse, with or without the use of phosphodiesterase type 5 inhibitors. Postoperative complications were classified according to the Clavien-Dindo system.

Inclusion and exclusion criteria

We included patients with localized adenocarcinoma of the prostate diagnosed after transrectal ultrasound-guided biopsy of the prostate [7]. All patients were staged with multiparametric magnetic resonance imaging (mp-MRI) with no previous history of radiation therapy or surgical treatment for benign hyperplasia. We did not include patients with incomplete perioperative data.

Data acquisition

Data acquisition was conducted following approval from the ethics committee. This included age, prostatic volume by MRI, PSA levels, comorbidities, postoperative complications, histopathology results, erectile function, continence status, and biochemical recurrence data.

Surgical technique

All patients underwent general anesthesia and were placed in the Trendelenburg and lithotomy position with protection pads in the extremities and compression stockings. The pneumoperitoneum was performed through an upper umbilical incision with an open technique. A total of six ports were placed (four robotics and two assisted); subsequently, transperitoneal posterior RRP was performed [8]. The following are the surgical steps followed: (1) Douglas pouch incision, (2) athermal seminal vesicle dissection, (3) Denonvillier’s fascia identification and dissection throughout the prostate apex, (4) Retzius space dissection, (5) dorsal venous complex ligation, (6) endopelvic fascia dissection, and (7) anterior and posterior bladder neck incision.

Antegrade dissection and preservation of the neurovascular bundle was performed when indicated, following a standard interfascial plane approach. Dissection was performed using athermal techniques, mainly with Hem-o-lok clips for vascular control. Nerve-sparing (unilateral or bilateral) was decided based on tumor location and intraoperative assessment.

Pelvic lymphadenectomy was performed in intermediate and high-risk cases. Continuous anastomosis was performed in all cases according to the Van Velthoven technique (20-Fr Foley catheter) [9]. At the end of the surgery, an abdominal drain was placed. After admission to the post-anesthetic care unit, all patients were transferred to the standard hospitalization room. All patients were included in thromboembolic prevention protocols [10]. All patients were advised to take tadalafil 5 mg daily for corporal rehabilitation and were encouraged to perform frequent pelvic floor (Kegel) exercises during the recovery period to enhance continence and potency. These measures were recommended as general guidance and were not part of a structured or supervised rehabilitation program. Patients were followed regularly to assess urinary and sexual function and guide ongoing care.

Statistical analysis

Categorical variables were reported as counts and percentages. Continuous variables were summarized using the median, range, and interquartile range (IQR). Associations between pathological stage and biochemical recurrence, as well as between positive surgical margins and biochemical recurrence, were analyzed using Fisher’s exact test. Biochemical recurrence-free survival was analyzed using the Kaplan-Meier method. All statistical analyses were performed using JASP 0.95.4 (Apple Silicon, Cupertino, CA, USA) software.

## Results

A total of 87 patients underwent RRP; however, only 70 patients were included in the analysis due to incomplete data for the remaining 17 patients.

Baseline and perioperative characteristics

The median age was 64 years, PSA was 4.48 ng/dL, and prostate volume was 40 cc. Overall, 31% of patients had hypertension, and 12.85% had type 2 diabetes mellitus. Only one case was converted to open surgery, and no blood transfusions were necessary (Table 1).

**Table 1:** Epidemiological and perioperative data. IQR = interquartile range; PSA = prostatic-specific antigen; ISUP: International Society of Urological Pathology

Perioperative characteristics	IQR	Median	Range
Age (years)	59–69	64	46–75
Prostate size (cc)	30–54	40	18–114
PSA (ng/mL)	4.03–8.48	4.98	1–64
Blood transfusion	None	None	None
Preoperative pathology			
ISUP 1, n (%)	30 (42.86%)	-	-
ISUP 2, n (%)	18 (25.71%)	-	-
ISUP 3, n (%)	18 (25.71%)	-	-
ISUP 4, n (%)	4 (5.71%)	-	-
ISUP 5, n (%)	0	-	-
Conversion rate, N (%)	1 (1.42%)	-	-

During the immediate postoperative period, complications were observed in 5 out of 71 (7.04%) patients. Among these complications, three (4.2%) cases were classified as Clavien III. These Clavien III complications were attributed to urinary leakage from the anastomosis. However, all three cases were successfully managed with endoscopic intervention and replacement of the Foley catheter (Table 2).

**Table 2:** Follow-up data and complications. DVT = deep venous thrombosis; IQR = interquartile range

	N (%)	Complication
Clavien-Dindo grade		
I	1 (1.42%)	Fever
II	1 (1.42%)	DVT
IIIa	0	-
IIIb	3 (4.2%)	Urinary leak
IVa	0	-
IVb	0	-
V	0	-
Data		
Median follow-up	38	-
IQR	25–40	-
Patients followed	62/70	-
Continence rate, n (%)	58 (93.54%)	-
Potency rate, n (%)	41 (66.12%)	-
Biochemical recurrence rate, n (%)	12/62 (19.35%)	-

Follow-up and functional outcomes

The most common preoperative pathology was International Society of Urological Pathology (ISUP 1) with a rate of 43% (Table 1). The definitive pathology analysis reported an increase in the ISUP 2 group (50%). The positive margin rate was 22.85% (16 cases) (11.11% of all pT2 and 44% of all pT3), as shown in Table 3.

**Table 3:** Postoperative pathology characteristics. ISUP = International Society of Urological Pathology; PSM: positive surgical margins

	N (%)
ISUP grade	
ISUP 1	14 (20%)
ISUP 2	35 (50%)
ISUP 3	13 (18.57%)
ISUP 4	4 (5.71%)
ISUP 5	4 (5.71%)
Pathology report	
pT2a	10 (14.28%)
pT2b	12 (17.14%)
pT2c	23 (32.85%)
PT3a	18 (25.71%)
pT3b	7 (10%)
PSM	
pT2	5 (11.11%)
pT3	11 (44%)

The mean follow-up was 38 months, and a total of eight patients were lost during follow-up. Of the 62 patients, 93.54% achieved complete continence after one year from surgery, 66.12% could maintain satisfactory erections, and 19.35% experienced biochemical recurrence (Table 2).

Association between pathological characteristics and biochemical recurrence

**Figure 1:**
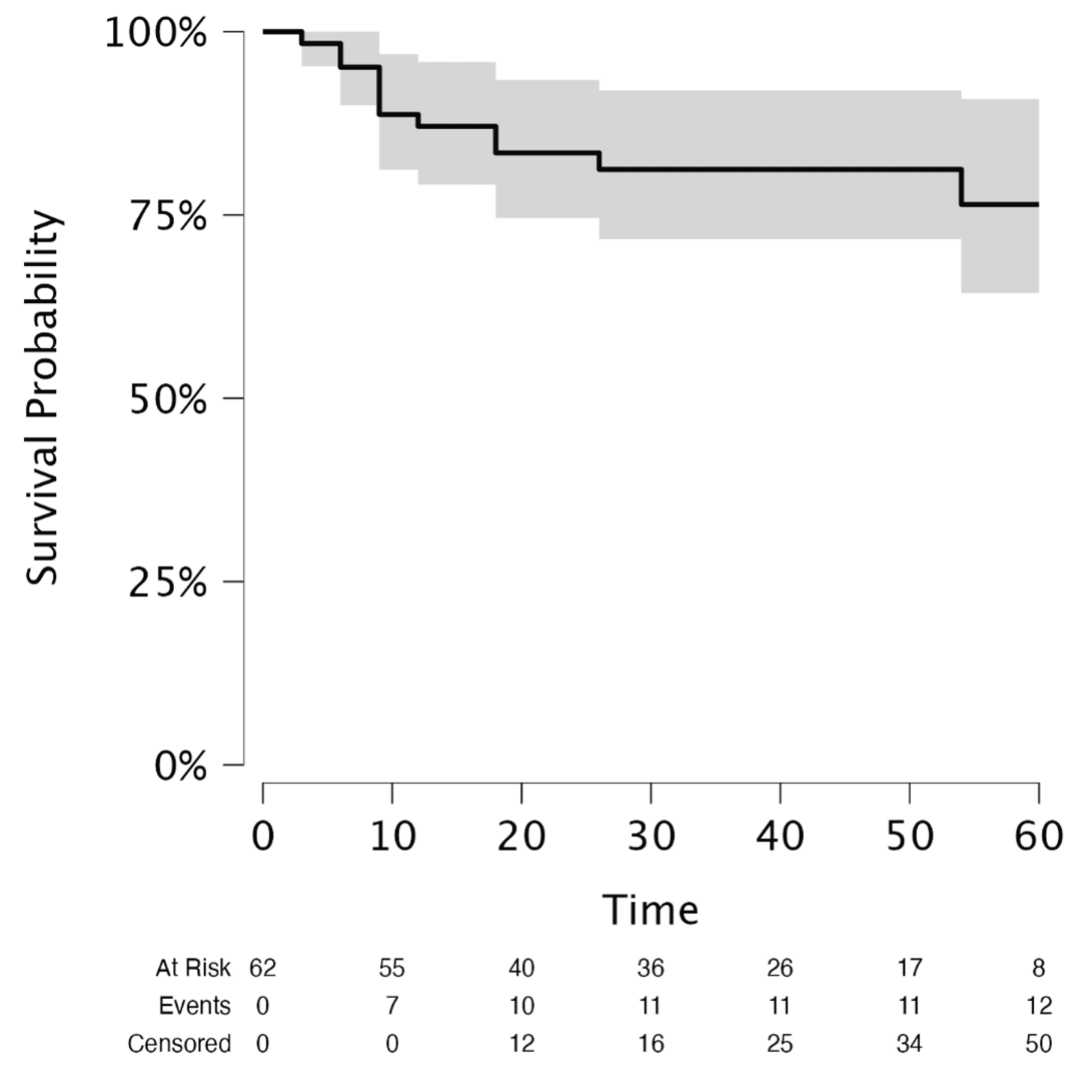
Kaplan-Meier curve for biochemical recurrence-free survival. Survival probability: biochemical-free survival probability. Time: time to biochemical recurrence (months).

During a mean follow-up of 38 months, 12 out of 62 (19.35%) patients experienced biochemical recurrence. Kaplan-Meier analysis demonstrated a restricted mean survival time of 50.7 months (standard error = 2.47; 95% confidence interval (CI) = 45.9-55.5), reflecting the time to biochemical recurrence for the cohort (Figure 1).

Analysis of the association between surgical margin status and biochemical recurrence using contingency tables and Fisher’s exact test showed that patients with positive surgical margins had a significantly higher risk of biochemical recurrence compared to those with negative margins (10/16 (62.5%) vs. 2/46 (4.3%); odds ratio (OR) = 3.60, 95% CI = 1.86-5.34; p < 0.001).

Similarly, the comparison between pathologic stages revealed that patients with pT3 disease had a higher biochemical recurrence rate than those with pT2 disease (11/23 (47.8%) vs. 1/39 (2.6%); OR = 3.55, 95% CI = 1.40-5.70; p < 0.001). These findings were consistent with the Kaplan-Meier curves, which showed shorter biochemical recurrence-free survival in patients with positive margins and advanced pathologic stage.

## Discussion

Regarding oncological outcomes, we observed a positive surgical margin rate of 22.85%, with 11.11% of all pT2 cases and 44% of all pT3 cases affected. Similar findings were reported in the literature from Latin America, where Rocha et al. documented a positive surgical margin rate of 21.81%, affecting 16.27% of all pT2 cases and 45.45% of all pT3 cases [11]. Additionally, other studies from Latin America have reported positive surgical margin rates ranging from 18% to 21.6% [12,13].

In other international reports, Cao et al. conducted a systematic review and meta-analysis, reporting a positive surgical margin rate of 22.3%, with rates of 18.8% and 41.4% for pT2 and pT3 cases, respectively [14]. Coelho et al. reported in a review of current outcomes in 2009 a positive surgical margin rate of 15%, with rates of 9.4% and 50% for pT2 and pT3 cases, respectively [15]. Hu et al. reported an overall positive surgical margin rate of 13.6%, with rates of 10.3% and 28.8% for pT2 and pT3 cases, respectively [16].

We achieved complete continence and satisfactory erections in 93.54% and 66.12% of patients, respectively. In the literature, including results from Latin America, continence rates after 6 and 12 months ranged from 70.8% to 94% [11,13-15]. Potency reports after 6 and 12 months included rates of 70% [11,13], 71.2% [15], and 69.2% [16].

This study has limitations, starting with its retrospective nature and the loss of eight (11.42%) patients during follow-up. Compared with other retrospective analyses, we did not register operating time because of the inconsistency of the register. The use of questionnaires like the International Index of Erectile Function or the Sexual Health Inventory for Men before and after surgery would have been more accurate and objective when reporting results; unfortunately, it was difficult to implement this for all patients because some continued their follow-up in other centers [17]. We could not include other patients in the study because of a significant lack of data during their follow-up. As some patients were from different regions of the country, they continued their follow-up in their regional hospital after surgery.

Despite our limitations, to our knowledge, this represents the inaugural historical publication of robotic surgery results from Uruguay. Our efforts will focus on minimizing patient follow-up loss and further developing our database registry.

## Conclusions

Despite being a low-volume center, the initial experience with robotic-assisted radical prostatectomy in Uruguay demonstrated satisfactory perioperative, oncologic, and functional outcomes. These results appear to be within the range reported in the international literature, particularly in the context of proctor-led procedures. However, larger prospective studies with standardized outcome measures are needed to confirm these findings and better define the impact of surgical experience on outcomes.
